# Preterm Infant and Caregiver Outcomes After Maternal Appendectomy During Pregnancy

**DOI:** 10.3390/healthcare14131822

**Published:** 2026-06-23

**Authors:** Sergiu Costescu, Adrian Ratiu, Danut Dejeu, Oana Cristina Costescu, Daniela Mariana Cioboata, Denis Gruber, Ioana Mihaela Citu, Cosmin Citu

**Affiliations:** 1 Doctoral School, “Victor Babes” University of Medicine and Pharmacy, Eftimie Murgu Square 2, 300041 Timisoara, Romania; costescu.sergiu@umft.ro (S.C.); denis.gruber@umft.ro (D.G.); 2 Department of Obstetrics and Gynecology, “Victor Babes” University of Medicine and Pharmacy, Eftimie Murgu Square 2, 300041 Timisoara, Romania; ratiu.adrian@umft.ro (A.R.); citu.ioan@umft.ro (C.C.); 3 Surgical Oncology Department, Emergency County Hospital Oradea, 410169 Oradea, Romania; 4 Discipline of Neonatology, Faculty of Medicine, “Victor Babes” University of Medicine and Pharmacy, Eftimie Murgu Square 2, 300041 Timisoara, Romania; cioboata.daniela@umft.ro; 5 Department of Internal Medicine I, “Victor Babes” University of Medicine and Pharmacy, Eftimie Murgu Square 2, 300041 Timisoara, Romania; citu.ioana@umft.ro

**Keywords:** appendectomy, caregiver burden, infant, premature, intensive care units, neonatal, neonatal sepsis

## Abstract

Background and Objectives: Appendectomy during pregnancy is associated with preterm birth, but downstream neonatal outcomes, neonatal intensive care resource use, and caregiver-reported psychological symptom burden remain insufficiently characterized. We aimed to compare neonatal infection rates, NICU resource utilization, and caregiver psychosocial outcomes between preterm infants born after maternal appendectomy during pregnancy and preterm controls frequency-matched by gestational-age strata without antecedent non-obstetric surgery. Methods: In this single-center prospective cohort study (March 2023–December 2025), 121 preterm infants were enrolled: 54 born after maternal appendectomy during pregnancy (31 laparoscopic, 23 open) and 67 non-surgical preterm controls. Neonatal outcomes included culture-confirmed infection, death, or major neonatal morbidity, and neonatal intensive care resource metrics. Caregiver outcomes were assessed near discharge using the 36-Item Short Form Survey, Patient Health Questionnaire-9, Generalized Anxiety Disorder-7 scale, and Hospital Anxiety and Depression Scale. Group comparisons used normality-guided parametric or non-parametric tests and multivariable logistic regression; subgroup and mediation analyses were exploratory. Mediation analyses explored indirect pathways. Results: Culture-confirmed infection was numerically more frequent in appendectomy-group neonates than in controls (35.2% versus 20.9%; *p* = 0.078), but this difference was not statistically significant. NICU length of stay was significantly longer (47.3 ± 14.8 vs. 41.2 ± 12.6 days; *p* = 0.014), and caregiver Patient Health Questionnaire-9 depressive symptom scores were higher (12.4 ± 4.3 vs. 9.6 ± 3.8; *p* < 0.001). Open appendectomy and negative histopathology subgroups showed the strongest adverse signals. Exploratory mediation analysis suggested that a substantial portion of the appendectomy-caregiver depression association statistically co-varied with prolonged hospitalization (Sobel *p* = 0.008); this exploratory pathway analysis does not establish a causal mediation pathway. Conclusions: Preterm infants born after maternal appendectomy during pregnancy showed non-significant numerical increases in infection outcomes, significantly higher neonatal intensive care resource use, and higher caregiver-reported psychological symptom scores compared with non-surgical preterm controls, with open surgery and negative appendectomy representing clinically complex subgroups with less favorable exploratory signals.

## 1. Introduction

Preterm birth remains a leading driver of neonatal mortality and long-term neurodevelopmental, respiratory, and metabolic morbidity worldwide [1,2]. Among the heterogeneous causes of prematurity, non-obstetric surgical emergencies during pregnancy occupy a distinctive pathophysiologic niche, because both the inflammatory burden of the underlying abdominal disease and the physiologic stress of surgery may precipitate uterine activity, alter placental function, and increase the likelihood of medically indicated or spontaneous preterm delivery [3,4,5,6]. Acute appendicitis is the most common non-obstetric surgical emergency in pregnancy, and appendectomy remains the accepted treatment when the diagnosis is considered likely after clinical and imaging evaluation [3,7].

Contemporary evidence has established that appendectomy during pregnancy is associated with a measurable increase in preterm birth risk [3,4,5,6,7]. Population-based and cohort data have reported adjusted risk estimates in the range of approximately 1.7–2.2 for preterm delivery after appendectomy, with the magnitude of risk influenced by surgical approach, gestational timing, perforation status, and whether histopathology confirms true appendicitis or a negative appendectomy [3,4,5,6,7]. These observations suggest that both the disease process and the operative response can shape downstream obstetric outcomes.

While the obstetric sequelae of appendectomy during pregnancy have been increasingly studied, the neonatal trajectory of preterm infants born in this context remains largely unexamined. Most available reports focus on whether preterm birth occurred rather than on the postnatal course of the infant. This is a clinically important knowledge gap, because preterm neonates exposed to maternal surgical and inflammatory stress may differ from other preterm infants in baseline immune activation, susceptibility to infection, and subsequent organ-specific morbidity [6,7].

In the neonatal intensive care setting, culture-confirmed infection—whether bacterial sepsis or invasive candidiasis—is a trajectory-changing event associated with substantially higher risks of bronchopulmonary dysplasia, severe brain injury, prolonged hospitalization, and excess mortality in very preterm infants [8,9,10,11]. In parallel, the NICU course exerts major psychosocial pressure on caregivers, and parental anxiety, depression, and impaired quality of life are consistently reported when infants require prolonged intensive care [12]. These two domains, neonatal infection burden and caregiver distress, are therefore likely to intersect in infants born preterm after maternal appendectomy during pregnancy.

Data remain limited regarding whether the specific etiology of preterm birth—maternal appendectomy versus non-surgical causes—is associated with neonatal infection patterns, neonatal intensive care resource utilization, and caregiver-reported psychological outcomes. If maternal appendectomy identifies a subgroup of premature infants with a more resource-intensive postnatal course, this will support targeted surveillance, intensified infection-prevention strategies, and earlier psychosocial support for families [3,4,5,6,7,12].

In addition, caregiver outcomes after a pregnancy complicated by emergency surgery may reflect both the infant’s neonatal intensive care course and the mother’s own perioperative experience, postoperative recovery, obstetric complications, social support, and pre-existing mental health status. This makes cautious interpretation important when family-centered outcomes are studied in this setting.

The objective of this study was to compare culture-confirmed infection, neonatal intensive care resource utilization, major neonatal morbidity, and caregiver-reported psychological outcomes between preterm infants born after maternal appendectomy during pregnancy and frequency-matched, gestational-age-comparable preterm infants born without antecedent non-obstetric surgery. Secondarily, we aimed to explore whether appendectomy-specific characteristics, including surgical approach, histopathological findings, trimester at surgery, and surgery-to-delivery interval, were associated with differential neonatal and caregiver outcomes. Because of the observational design and modest subgroup sizes, analyses of surgical approach, histopathology, trimester, mediation, and time-to-event patterns were considered exploratory and hypothesis-generating.

## 2. Materials and Methods

### 2.1. Study Design, Setting, and Case Ascertainment

This investigation was structured as a single-center prospective observational cohort study conducted within the Neonatology and Neonatal Intensive Care Unit affiliated with the “Victor Babes” University of Medicine and Pharmacy, Timisoara, Romania. Enrollment occurred from March 2023 through December 2025, and eligible preterm infants admitted to the unit were screened prospectively for inclusion according to predefined exposure and control criteria. The study was designed and reported as an observational comparative cohort with predefined exposure groups and prospectively collected outcome data [6,7]. The institution functions as a tertiary maternal-fetal and neonatal referral center receiving inborn cases and selected regional transfers; therefore, the present sample should be interpreted as an exposure-enriched comparative cohort rather than a population denominator study of all pregnancies complicated by appendectomy. To assist readers in gauging the referral context, approximate institutional activity during the 34-month enrollment window included roughly 4500–5000 annual deliveries, approximately 750–850 annual NICU admissions, and an estimated 180–220 live births per year before 34 completed weeks of gestation, with an inborn-to-outborn ratio of approximately 3:1 among very preterm admissions. These figures are administrative approximations rather than prospectively audited denominators; because mothers with appendectomies during pregnancy were preferentially referred to and delivered at this tertiary center, the exposed group is enriched relative to a general obstetric population, and the resulting potential for selection and referral bias is discussed further in the Limitations.

The study protocol was developed in accordance with the principles of the Declaration of Helsinki and current Good Clinical Practice guidelines. Ethical approval was obtained from the Local Commission of Ethics of the Clinical County Hospital of Timișoara, Romania (approval number E-1047, date of approval: 14 February 2023). Written informed consent was obtained from caregivers of all enrolled neonates prior to data collection, covering both clinical chart abstraction and the administration of caregiver-reported outcome instruments. Because caregiver mental health instruments were incorporated into the protocol, a predefined referral pathway was established for caregivers whose screening scores exceeded predefined clinical thresholds, consistent with current recommendations for identifying parental distress in the NICU setting [12].

### 2.2. Participants, Eligibility, and Group Definitions

A total of 121 preterm infants were enrolled and divided into two groups based on maternal surgical exposure during the index pregnancy. The appendectomy group (*n* = 54) comprised preterm neonates whose mothers underwent appendectomy (laparoscopic or open) for suspected or confirmed appendicitis at any gestational age during the pregnancy that resulted in the index preterm birth. The control group (*n* = 67) consisted of preterm neonates admitted to the same NICU during the study period whose mothers had no history of non-obstetric abdominal surgery during the index pregnancy. Controls were selected from eligible contemporaneous NICU admissions without antecedent non-obstetric abdominal surgery and were frequency-matched on gestational-age strata (≤28 weeks, 29–31 weeks, 32–33 weeks), not individually matched, to ensure broad comparability in prematurity severity across groups [3,6]. Because multiple gestations were eligible, the 121 analyzed neonates were born to 116 mothers (111 singleton pregnancies and 5 twin pregnancies), corresponding to a pregnancy-to-neonate ratio of approximately 1:1.04. In the appendectomy group, 54 neonates were born to 52 mothers (2 twin pregnancies), and in the control group, 67 neonates were born to 64 mothers (3 twin pregnancies). Family-level clustering arising from multiple gestations was addressed in sensitivity analyses, and caregiver-reported instruments were completed once per family to avoid duplicate family-level observations.

For controls, the reason for preterm birth was abstracted when available from obstetric records and included spontaneous preterm labor, premature rupture of membranes, hypertensive disorders, placental disease, fetal growth restriction, and suspected intra-amniotic infection. These pathways were not used to create separate powered strata because of sample-size constraints.

Appendectomy-exposed cases were identified through maternal surgical records, obstetric records, and neonatal admission documentation. The study did not prospectively capture the full institutional denominator of all deliveries, all maternal appendectomies during pregnancy, or all <34-week births during the study period; this was added as a limitation because referral patterns may have enriched the exposed cohort.

Eligibility criteria for both groups required birth at less than 34 completed weeks of gestation, NICU admission within the first 24 h of life, and a minimum NICU observation period of seven days when survival and transfer status allowed outcome ascertainment. Exclusion criteria were major congenital anomalies incompatible with survival, chromosomal abnormalities, transfer to another facility before complete outcome capture, and cases in which the maternal surgical history could not be confirmed through medical records. For infants with multiple infection episodes during the NICU stay, infection status was classified according to the first culture-confirmed episode, consistent with commonly used neonatal sepsis surveillance frameworks [8,9,10]. Infants who died before seven days were retained for mortality and composite-outcome ascertainment, whereas infants discharged or transferred before complete infection surveillance were excluded; the potential for selection bias from this criterion is acknowledged in the Limitations.

### 2.3. Data Collection and Outcome Measures

Clinical data were abstracted from NICU records using a standardized case report form developed for this study. Baseline infant variables included gestational age at birth, birth weight, sex, multiplicity, small-for-gestational-age status, mode of delivery, antenatal steroid exposure, premature rupture of membranes, clinical chorioamnionitis, and early illness severity quantified using the SNAPPE-II within the first 12 h of life. Caregiver-reported outcomes were assessed within 72 h before planned discharge using validated local-language versions of the SF-36, WHOQOL-BREF, HADS, PHQ-9, and GAD-7 [11,12,13,14,15]. The respondent was the mother whenever available; if the mother was unavailable, the primary caregiver who participated most consistently in discharge planning completed the questionnaires. When both parents were present, only one predefined primary-caregiver response was analyzed to avoid duplicate family-level observations. The Body Image Scale was included to capture perceived body-image concerns after pregnancy, surgery, and prolonged hospitalization; higher scores indicate greater concerns.

The primary neonatal outcome was culture-confirmed infection during the NICU stay, defined as at least one positive blood culture for a recognized bacterial pathogen or *Candida* species in the context of compatible clinical signs and targeted antimicrobial treatment. Early-onset sepsis was defined as infection occurring before 72 h of life, and late-onset sepsis as infection occurring at or after 72 h of life; infections were further classified by organism type. Secondary neonatal outcomes included a composite adverse outcome defined as death or any major morbidity, together with NICU resource metrics such as length of stay, invasive ventilation days, central-line days, antimicrobial exposure, and transfusion burden. These outcome domains were selected because they align with the established clinical burden of neonatal sepsis and invasive candidiasis in very preterm populations [8,9,10,11]. Coagulase-negative staphylococci were classified as true infection only when accompanied by clinical deterioration and antimicrobial treatment; repeated positive cultures, central-line status, and inflammatory markers were considered when available to distinguish true infection from contamination. Candidemia was defined as *Candida* species growth from blood culture, and recurrent infection was defined as two or more temporally distinct culture-confirmed episodes after completion or de-escalation of treatment for the prior episode.

The composite adverse neonatal outcome was defined a priori as death before hospital discharge or at least one major neonatal morbidity. Components were moderate-to-severe bronchopulmonary dysplasia assessed at 36 weeks postmenstrual age or discharge, necrotizing enterocolitis Bell stage II or higher, severe intraventricular hemorrhage grade III–IV on cranial ultrasound, retinopathy of prematurity requiring treatment according to ophthalmologic criteria, and periventricular leukomalacia diagnosed by cranial ultrasound or magnetic resonance imaging when clinically performed.

### 2.4. Statistical Analysis

All analyses were conducted using SPSS version 28.0 (IBM Corporation, Armonk, NY, USA) and R version 4.3.1. Continuous variables were inspected graphically and assessed for normality using the Shapiro–Wilk test; normally distributed variables were expressed as mean ± standard deviation and compared using independent-samples *t*-tests or Welch tests, whereas non-normally distributed variables were compared using Mann–Whitney U tests. Categorical variables were compared using Pearson’s chi-squared test or Fisher’s exact test. Multivariable logistic regression was used to model the composite adverse neonatal outcome, and mediation analyses were performed with bootstrap resampling to explore indirect pathways between surgical exposure, neonatal burden, and caregiver psychological distress, and were interpreted as exploratory. Caregiver scale scoring followed the standard published frameworks for the SF-36, WHOQOL-BREF, HADS, PHQ-9, and GAD-7 [11,12,13,14,15]. Analyses were classified as primary, secondary, or exploratory. The primary analysis was the comparison of culture-confirmed infection between groups; secondary analyses included composite morbidity, resource use, and caregiver scale scores; subgroup, mediation, survival, and figure-based analyses were exploratory. Missing clinical variables were rare and were handled using complete-case analysis; incomplete caregiver questionnaires were excluded from the corresponding scale analysis. Because multiple births were included, sensitivity checks using family-level clustering were considered when applicable. Standardized mean differences were reviewed descriptively for baseline balance in addition to *p*-values.

Multivariable logistic regression was used to model the composite adverse neonatal outcome, with covariates selected a priori based on clinical relevance and constrained by the number of outcome events: group assignment (appendectomy versus control), gestational age, SNAPPE-II score, culture-confirmed infection, sex, and exploratory appendectomy-specific indicators. Because the composite adverse outcome occurred in only 38 infants, the number of events per variable (EPV) was low, and inclusion of all candidate predictors in a single model risked overfitting and unstable estimates. To address this, the regression was restructured into two models. A simplified main model, applied to the entire cohort, retained only the most clinically essential predictors that are defined identically in both groups (group assignment, gestational age, SNAPPE-II score, and culture-confirmed infection), keeping the EPV closer to the conventional rule-of-thumb threshold. The appendectomy-specific indicators (open versus laparoscopic approach, negative versus inflamed histopathology, and surgery-to-delivery interval), which are undefined in controls and were previously coded as absent, were reserved for a separate exploratory model restricted to the appendectomy group (*n* = 54). This avoids conflating structurally group-specific variables with the overall between-group comparison and confines those estimates to the population in which they are defined. Results were reported as adjusted odds ratios (aOR) with 95% confidence intervals (CI). Model fit was assessed using the Hosmer-Lemeshow goodness-of-fit test and the area under the receiver operating characteristic curve. To explore indirect pathways between exposures and outcomes, formal mediation analyses were conducted using bootstrap resampling (5000 resamples) and the Sobel test for indirect effects, but these analyses were considered hypothesis-generating. Specifically, we tested whether NICU resource burden (length of stay, ventilation days) mediated the relationship between (a) appendectomy exposure and caregiver psychological distress, and (b) infection status and caregiver outcomes. Pearson and Spearman correlation coefficients were used to quantify bivariate associations between continuous variables where appropriate. Interaction terms between surgical approach and infection status on NICU length of stay were tested in linear regression models. Variables defined only among appendectomy-exposed pregnancies were coded as absent in controls and interpreted only as exploratory markers; the overall group comparison and appendectomy-restricted subgroup analyses were therefore interpreted separately. Culture-confirmed infection may lie on the pathway between prenatal exposure and later neonatal morbidity, so models including infection were interpreted as adjusted associations rather than total causal effects. For time-to-first infection, Kaplan–Meier curves and Cox models were used through 60 days; infants without infection were censored at discharge or day 60, and death before infection was treated as a censoring event in the primary display, recognizing that competing-risk methods may be preferable in larger cohorts. Because the appendectomy group had a longer mean length of stay, infants in that group remained under infection surveillance for a longer period; this differential observation time can increase the number of detected infections and the apparent cumulative incidence independently of the underlying hazard. We therefore interpreted the log-rank and Cox results as exploratory and potentially confounded by length of stay. Discharge before infection was handled as administrative censoring, and pre-infection death was treated as censoring in the primary display; because treating death as censoring can overestimate cumulative infection incidence when mortality differs between groups, a competing-risks (cumulative incidence function) framework would be preferable, and the absence of such analysis is acknowledged as a limitation. The time-to-event findings should be read in conjunction with the non-significant primary infection comparison rather than as independent confirmatory evidence.

## 3. Results

Table 1 presents the baseline neonatal and perinatal characteristics of the 121 preterm infants enrolled in this study, stratified by maternal appendectomy exposure. The two groups were broadly comparable on most demographic and perinatal variables, supporting the adequacy of the frequency-matching strategy. Mean gestational age was slightly higher in the appendectomy group (31.4 ± 2.3 weeks) than in the control group (30.8 ± 2.1 weeks), though this difference was not statistically significant (*p* = 0.142). Birth weight followed a similar non-significant pattern (1612.3 ± 421.7 g vs. 1547.8 ± 398.4 g; *p* = 0.389). Sex distribution, mode of delivery, antenatal steroid exposure, multiple gestation, small-for-gestational-age status, 5 min Apgar score below 7, and surfactant administration were all comparable between groups (all *p* > 0.40). Premature rupture of membranes was numerically less frequent in the appendectomy group (13.0% vs. 23.9%), but spontaneous and medically indicated preterm birth pathways were not analyzed as separate powered groups, so this non-significant difference should not be interpreted as evidence for a specific delivery mechanism (*p* = 0.127). Clinical chorioamnionitis was somewhat more prevalent in the appendectomy group (20.4% vs. 11.9%; *p* = 0.203). SNAPPE-II scores, reflecting early illness severity, were similar (19.7 ± 6.8 vs. 18.4 ± 7.1; *p* = 0.317), indicating that initial physiologic severity was comparable despite the differing etiologies of prematurity.

Table 2 details the surgical and histopathological characteristics within the appendectomy group, comparing laparoscopic (LA, *n* = 31) and open (OA, *n* = 23) subgroups. Women who underwent laparoscopic appendectomy had surgery at an earlier mean gestational age (19.8 ± 7.6 weeks) compared with those who had open procedures (24.3 ± 8.9 weeks; *p* = 0.048), consistent with the clinical preference for laparoscopy in earlier gestation and a tendency toward open surgery when the gravid uterus complicates trocar placement or visualization in later pregnancy. The surgery-to-delivery interval was correspondingly longer in the laparoscopic group (97.3 ± 39.8 days vs. 72.1 ± 43.6 days; *p* = 0.028), reflecting both the earlier timing of surgery and potentially a lower inflammatory stimulus permitting longer gestational continuation. Histopathologically inflamed appendices predominated in both subgroups (74.2% LA vs. 65.2% OA; *p* = 0.468), and the overall negative appendectomy rate was 29.6% (16/54), with a non-significant trend toward more negative appendectomies in the open group (34.8% vs. 25.8%). Operative time was shorter for laparoscopic procedures (48.6 ± 14.3 vs. 57.9 ± 18.7 min; *p* = 0.043), and maternal postoperative hospital stay was significantly shorter after laparoscopy (2.8 ± 1.1 vs. 4.3 ± 1.9 days; *p* = 0.001).

Table 3 summarizes neonatal infection outcomes and microbiological profiles across the two study groups. The overall rate of culture-confirmed infection was numerically higher in the appendectomy group (35.2%, 19/54) than in controls (20.9%, 14/67), but this difference did not reach conventional statistical significance (*p* = 0.078). Bacterial sepsis was the predominant infection type in both groups (25.9% vs. 16.4%; *p* = 0.195), while candidemia was infrequent overall and numerically more common in the appendectomy group (9.3% vs. 4.5%; *p* = 0.465). Nearly all infections were late onset (≥72 h), and the mean day of life at first positive culture was earlier in the appendectomy group (11.4 ± 4.7 vs. 13.8 ± 5.1 days; *p* = 0.152), although this timing difference was not statistically significant. Coagulase-negative staphylococci and Gram-negative organisms were the most commonly isolated pathogens, without significant differences in organism distribution between groups. Recurrent infection (≥2 culture-confirmed episodes) was numerically more frequent in the appendectomy group (11.1% vs. 4.5%; *p* = 0.176), but this difference was not statistically significant. Overall, infection results are best interpreted as a directional, non-confirmatory signal rather than definitive evidence of increased infectious morbidity.

Table 4 presents the major neonatal clinical outcomes by study group. The composite adverse outcome occurred in 38.9% (21/54) of appendectomy-group neonates compared with 25.4% (17/67) of controls, a numerical difference of 13.5 percentage points that did not achieve statistical significance (*p* = 0.107). In-hospital mortality was comparable (7.4% vs. 9.0%; *p* = 0.758). Moderate-to-severe bronchopulmonary dysplasia was more frequent in the appendectomy group (29.6% vs. 20.9%; *p* = 0.265). Necrotizing enterocolitis Bell stage ≥ II was numerically higher (11.1% vs. 4.5%; *p* = 0.176). Severe intraventricular hemorrhage and retinopathy of prematurity requiring treatment were also more frequent in the appendectomy group (13.0% vs. 7.5% and 14.8% vs. 7.5%, respectively), but none of these individual comparisons reached statistical significance. Therefore, the composite outcome was treated as a non-significant numerical trend rather than a confirmed morbidity difference.

Table 5 quantifies the NICU resource utilization differences between the two groups, showing the most consistent between-group differences in the study. Length of stay was significantly longer in the appendectomy group (47.3 ± 14.8 vs. 41.2 ± 12.6 days; *p* = 0.014), representing a mean excess of 6.1 days per infant. Invasive mechanical ventilation was required for significantly more days (21.6 ± 9.7 vs. 17.8 ± 8.4 days; *p* = 0.022), and total respiratory support followed the same pattern (36.8 ± 15.1 vs. 31.4 ± 13.2 days; *p* = 0.036). Central-line days were also elevated (21.4 ± 10.3 vs. 17.6 ± 8.7 days; *p* = 0.028). Antimicrobial exposure was also greater: systemic antibiotic days averaged 16.4 ± 7.9 versus 13.2 ± 6.1 (*p* = 0.011), and antifungal days were more than doubled (3.1 ± 5.4 vs. 1.4 ± 3.2; *p* = 0.029).

Table 6 presents caregiver-reported psychological outcomes at the pre-discharge timepoint, stratified by neonatal study group. Caregivers of appendectomy-group infants reported less favorable scores across several measured domains. The SF-36 Mental Component Summary showed a significant decrement (39.2 ± 7.1 vs. 43.6 ± 7.8; *p* = 0.002). PHQ-9 depressive symptom scores averaged 12.4 ± 4.3 in the appendectomy group versus 9.6 ± 3.8 in controls (*p* < 0.001), which falls within the moderate depressive symptom range. GAD-7 anxiety symptom scores were similarly elevated (11.1 ± 3.6 vs. 8.9 ± 3.3; *p* = 0.001), as were both HADS subscales. Body image concerns, measured by the BIS, were also higher (8.7 ± 3.1 vs. 6.8 ± 3.4; *p* = 0.002). These findings indicate higher screening-based psychological symptom burden near discharge, without establishing a clinical psychiatric diagnosis or proving that maternal appendectomy directly caused caregiver distress. The respondent was the mother in 96 of 116 families (82.8%) and another primary caregiver (predominantly the father) in 20 families (17.2%); in the appendectomy group, the mother—who had undergone surgery—was the respondent in 44 of 52 families (84.6%), compared with 52 of 64 families (81.3%) in the control group. Because the respondent could be either the mother (whose scores may reflect her own perioperative and obstetric experience in addition to the infant’s course) or another caregiver, this distribution is relevant to interpretation and is now reported by group. The number of valid responses per scale and group, with missing data, is summarized in Table 6: completion ranged from 49 to 52 of 54 (90.7–96.3%) in the appendectomy group and 61–65 of 67 (91.0–97.0%) in controls, with item-level missingness handled by complete-case analysis for each scale. A sensitivity analysis restricted to mother-respondents yielded score differences materially unchanged in direction and significance.

Table 7 presents the results of a multivariable logistic regression model examining variables associated with the composite adverse neonatal outcome. The model showed acceptable discriminative ability (AUC = 0.812) and adequate calibration (Hosmer–Lemeshow *p* = 0.384). Culture-confirmed infection was the strongest adjusted clinical predictor (aOR 3.27, 95% CI 1.41–7.58; *p* = 0.006). In exploratory appendectomy-specific coding, negative appendectomy showed the highest point estimate (aOR 3.41, 95% CI 1.18–9.86; *p* = 0.023), followed by open surgical approach (aOR 2.89, 95% CI 1.12–7.46; *p* = 0.028). Gestational age was inversely associated with the outcome (aOR 0.71 per additional week; *p* = 0.001). The surgery-to-delivery interval showed a modest inverse association (aOR 0.82 per 30-day increment; *p* = 0.041). Because the number of events was limited, these adjusted estimates should be viewed as exploratory and potentially sensitive to model specification. To respect the limited number of events (38 composite events), the estimates are now organized into two components. The simplified main model, applied to the full cohort, comprised only group assignment, gestational age, SNAPPE-II score, and culture-confirmed infection; in this model, culture-confirmed infection (aOR 3.27, 95% CI 1.41–7.58) and lower gestational age remained the dominant predictors, and the appendectomy-versus-control term was not statistically significant (aOR 2.14, 95% CI 0.96–4.78; *p* = 0.063). The appendectomy-specific indicators (open surgical approach, negative histopathology, and surgery-to-delivery interval) are reported only from the separate exploratory model restricted to the appendectomy group, because these variables are undefined in controls; their estimates are presented for hypothesis generation and are not part of the whole-cohort comparison. In Table 7, predictors belonging to the appendectomy-restricted exploratory model are indicated as such, and readers should not interpret the full set of coefficients as arising from a single overfitted model.

Table 8 provides a detailed subgroup analysis examining how specific appendectomy characteristics were associated with infection rates, composite adverse outcomes, NICU length of stay, and caregiver depressive symptom scores relative to the non-surgical control group. The open appendectomy subgroup showed numerically higher values for infection rate (43.5%), composite adverse outcome rate (47.8%), longest mean length of stay (52.1 ± 15.6 days), and highest caregiver PHQ-9 score (14.1 ± 4.3), with an exploratory interaction term (*p* = 0.037). Similarly, the negative appendectomy subgroup showed numerically less favorable outcomes across all metrics (infection 43.8%, composite 50.0%, LOS 52.4 ± 16.1, PHQ-9 13.9 ± 4.4; interaction *p* = 0.024). Third-trimester surgery showed the highest numerical infection rate (47.1%), composite adverse outcome (52.9%), and the most elevated caregiver distress (PHQ-9 14.6 ± 4.1; interaction *p* = 0.008). These subgroup estimates are hypothesis-generating because the surgical approach was related to gestational timing and clinical selection, and the subgroup sample sizes were small.

Table 9 presents exploratory mediation analyses evaluating whether NICU resource burden may partly explain the relationships between surgical and infection exposures and downstream outcomes. The largest exploratory indirect pathway suggested that nearly half (47.7%) of the total effect of appendectomy on caregiver PHQ-9 depression scores was mediated through prolonged NICU length of stay (indirect effect ab = 1.34; Sobel *p* = 0.008). A similar pattern was observed for SF-36 mental health, where 39.4% of the decline was mediated through increased ventilation days (Sobel *p* = 0.019). These percentages describe the proportion of the statistical association that co-varied with the candidate intermediate variable in an exploratory pathway model and should not be read as the proportion causally transmitted through that variable. Because length of stay, ventilation days, infection, central-line exposure, and caregiver symptom scores are temporally interrelated and subject to unmeasured confounding, the directionality assumed in these models cannot be confirmed in the present cohort. The infection-composite adverse outcome pathway suggested that 38.2% of the appendectomy effect on composite morbidity was mediated through culture-confirmed infection (Sobel, *p* = 0.031). Among infection-related exploratory pathways, 56.5% of the association between documented infection and caregiver HADS-Depression scores was mediated through length of stay (Sobel *p* = 0.003). These pathways should not be interpreted as causal proof because temporal ordering and unmeasured confounding cannot be fully resolved in this cohort.

**Figure 1:**
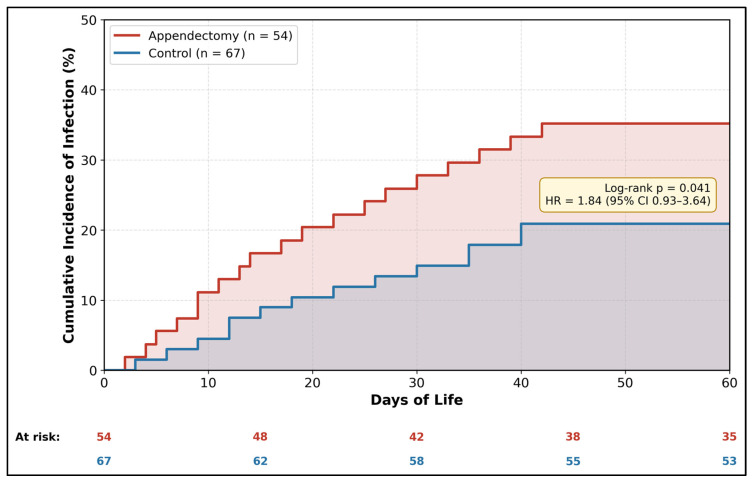
Cumulative incidence of first culture-confirmed neonatal infection over the first 60 days of life, by study group. Log-rank *p* = 0.041; overall HR 1.84 (95% CI 0.93–3.64). This time-to-event analysis is exploratory and potentially confounded. Because the appendectomy group had a significantly longer length of stay, its observation window for detecting infection was correspondingly longer, which can increase the apparent cumulative incidence independently of any true difference in infection hazard. Infants were censored at discharge or day 60, and death before infection was treated as a censoring event rather than as a competing risk; in the presence of differential discharge timing and pre-infection mortality, this approach may overestimate the cumulative incidence and should be interpreted alongside the non-significant primary infection comparison in Table 3 and the Cox confidence interval, which crossed 1.0.

Figure 1 illustrates the cumulative incidence of first culture-confirmed neonatal infection over the first 60 days of life. The appendectomy group showed a consistently higher cumulative incidence trajectory than controls, with separation evident from approximately day 8 of life and widening progressively, reaching approximately 35.2% at 60 days versus 20.9% in controls (log-rank *p* = 0.041; HR 1.84, 95% CI 0.93–3.64). The log-rank result and the Cox estimate were interpreted cautiously because the hazard-ratio confidence interval crossed 1.0, indicating limited precision.

**Figure 2:**
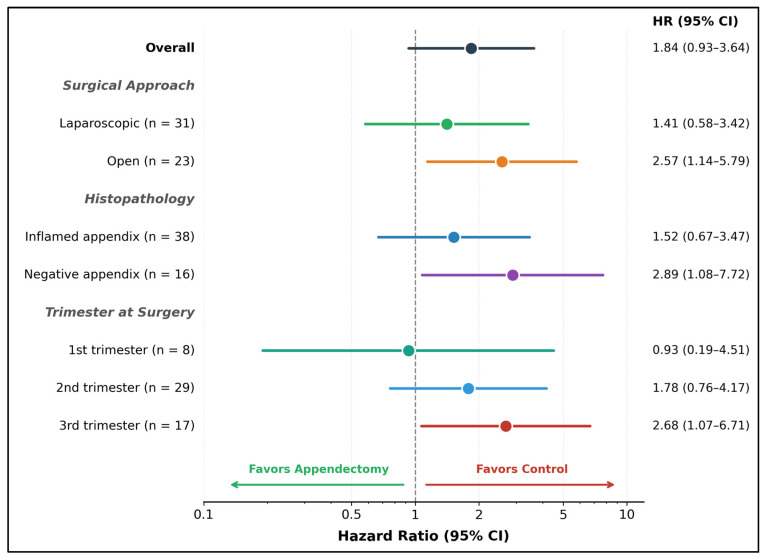
Subgroup hazard ratios for first culture-confirmed neonatal infection by appendectomy characteristics, compared with the non-surgical control group. Open appendectomy HR 2.57 (95% CI 1.14–5.79); negative appendectomy HR 2.89 (95% CI 1.08–7.72); third-trimester surgery HR 2.68 (95% CI 1.07–6.71). These subgroup hazard ratios are exploratory and hypothesis-generating only; they should not be interpreted as confirmatory evidence. Subgroup sizes were small, the subgroups were defined by characteristics related to gestational timing and clinical selection, and the estimates are subject to the same differential observation-time and confounding concerns described for Figure 1.

Figure 2 presents a forest plot of subgroup-specific hazard ratios comparing appendectomy subgroups with the non-surgical control group. The exploratory subgroup signal appeared strongest for the open appendectomy subgroup (HR 2.57, 95% CI 1.14–5.79) and the negative appendectomy subgroup (HR 2.89, 95% CI 1.08–7.72). Third-trimester surgery also showed a higher hazard estimate (HR 2.68, 95% CI 1.07–6.71), whereas first-trimester surgery showed no clear excess risk (HR 0.93, 95% CI 0.19–4.51).

**Figure 3:**
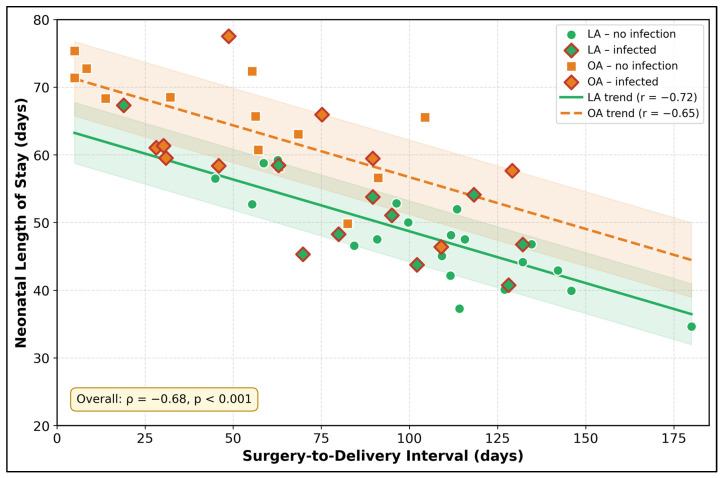
Surgery-to-delivery interval versus neonatal length of stay, stratified by surgical approach and infection status. Overall Spearman ρ = −0.68 (*p* < 0.001).

Figure 3 displays scatter plots with linear regression of the surgery-to-delivery interval against neonatal NICU length of stay, stratified by surgical approach. An unadjusted inverse correlation was observed overall (Spearman ρ = −0.68, *p* < 0.001), showing that shorter surgery-to-delivery intervals coincided with longer NICU stays. This relationship was visually similar for both laparoscopic (r = −0.72) and open (r = −0.65) approaches, but the open appendectomy regression line was shifted upward by approximately 5–8 days at comparable intervals. Because shorter intervals may also reflect earlier gestational age at delivery, birth weight, gestational age at surgery, and illness severity, this figure should be interpreted as descriptive rather than causal.

**Figure 4:**
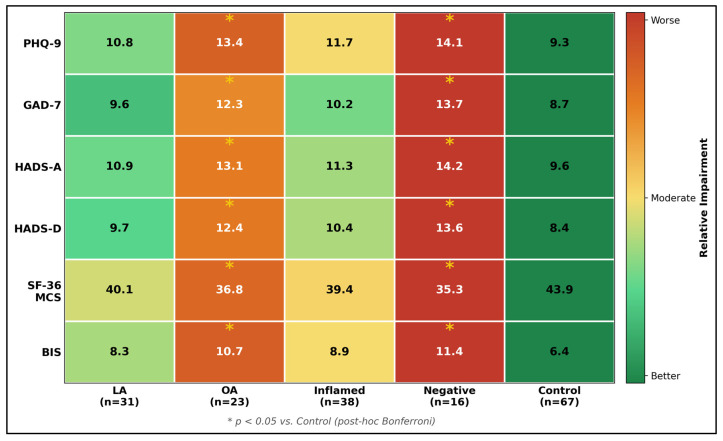
Caregiver psychological distress heatmap by appendectomy subgroup and control group. Asterisks indicate *p* < 0.05 versus control after Bonferroni correction.

Figure 4 presents a heatmap of caregiver standardized psychological and quality-of-life scores across appendectomy subgroups and the control group. The negative appendectomy and open appendectomy subgroups showed the least favorable color-coded profiles, including higher HADS-Anxiety, higher HADS-Depression, higher Body Image Scale scores, and lower SF-36 Mental Component Summary scores than controls (*p* < 0.05 after Bonferroni correction). Scale direction was harmonized for visualization so that warmer colors represented worse relative status; SF-36 MCS was reverse-coded for the heatmap only.

## 4. Discussion

### 4.1. Analysis of Findings

The present study provides an integrated analysis examining maternal appendectomy during pregnancy in relation to neonatal infection patterns, NICU resource utilization, and caregiver psychological outcomes in preterm infants. The numerically higher but non-significant infection proportions, together with longer hospitalization and greater respiratory and antimicrobial exposure observed in the appendectomy group, are biologically and clinically plausible within the broader neonatal sepsis literature, which has consistently shown that late-onset infection in very preterm infants is associated with prolonged hospital stay, higher mortality, and greater major morbidity [16,17,18,19,20,21]. At the same time, our exploratory subgroup findings suggest that this signal is not uniform: open surgery, negative appendectomy, and later gestational timing may represent clinically complex subgroups with less favorable exploratory signals, which is consistent with prior appendicitis-in-pregnancy literature reporting less favorable obstetric outcomes after negative or later procedures and potential advantages of minimally invasive approaches when clinically feasible [22,23,24,25,26,27,28,29,30]. The most robust findings of the present cohort relate to resource utilization and caregiver-reported symptom burden, whereas infection and composite morbidity results should be considered directional because the primary infection comparison did not reach conventional statistical significance.

The exploratory mediation analyses offer a possible framework for understanding these associations. The estimate that nearly half of the appendectomy-caregiver depression association statistically co-varied with prolonged NICU length of stay in an exploratory pathway analysis raises the hypothesis that interventions aimed at reducing avoidable hospitalization and device exposure could potentially provide dual benefit for infants and families. This cautious interpretation is consistent with prior neonatal studies demonstrating that late-onset sepsis, prolonged antibiotic exposure, and invasive support are linked to excess morbidity and longer admissions, thereby amplifying downstream family burden [16,17,18,19].

Our caregiver-reported symptom findings are also concordant with the broader NICU literature documenting high rates of parental anxiety, depression, post-traumatic symptoms, and reduced quality of life during and after neonatal hospitalization [20,21,25,26,27,28]. Importantly, the present analysis extends that literature by suggesting that the etiology of prematurity itself may be associated with caregiver psychological trajectories: families exposed first to an emergency maternal surgical event and then to a prolonged, complicated NICU course may report a compounded burden. This pattern supports considering routine screening, family-centered care models, and early psychosocial intervention for parents of high-risk preterm infants, particularly when maternal appendectomy, open surgery, or negative histopathology are part of the clinical history [20,21,25,26,27,28]. Because these associations are observational and the primary infection outcome was not statistically significant, this pattern is presented as a hypothesis for future evaluation rather than as a basis for changing current practice.

The baseline comparability shown in Table 1 is important for interpretation, because the two groups did not differ significantly in gestational age, birth weight, antenatal steroid exposure, or early illness severity (SNAPPE-II). This balance is consistent with population-based analyses indicating that appendectomy during pregnancy raises preterm birth risk without necessarily shifting infants into a more extreme prematurity stratum [3,4], and it supports attributing the observed downstream differences to factors other than gross prematurity severity alone. The surgical and histopathological profile in Table 2—earlier gestational age and longer surgery-to-delivery intervals for laparoscopic procedures, with open surgery clustered later in gestation—mirrors the practice patterns described in the SAGES guidance and in comparative series of laparoscopic versus open appendectomy in pregnancy, where laparoscopy is generally preferred earlier, and open approaches become more frequent as the gravid uterus enlarges [5,7]. The 29.6% negative-appendectomy rate is within the range reported for pregnancy, where diagnostic uncertainty and a low threshold for operation are well documented and where negative or later procedures have been linked to less favorable obstetric outcomes [4,30,31].

The infection findings (Table 3, Figure 1 and Figure 2) warrant cautious comparison with the literature. The numerically higher culture-confirmed infection proportion in the appendectomy group (35.2% vs. 20.9%) did not reach significance, and the predominance of late-onset rather than early-onset sepsis aligns with large network data showing that late-onset sepsis dominates the infectious burden of very preterm infants and is strongly associated with prolonged hospitalization and device exposure [8,9,16,17]. The hypothesis that maternal surgical and inflammatory stress could prime neonatal susceptibility to infection is biologically plausible given evidence linking intrauterine inflammation to altered neonatal immune activation [6,32]; however, our data cannot distinguish such priming from the confounding effect of longer observation time and greater central-line exposure in the appendectomy group, a point reinforced by the differential follow-up underlying Figure 1. The candidemia signal, although infrequent, is consistent with reports that invasive candidiasis tracks with central-line days and broad-spectrum antibiotic exposure in preterm infants [10,33], and it should be interpreted as a marker of cumulative device and antimicrobial burden rather than of exposure-specific risk.

The composite morbidity results (Table 4) and the regression in Table 7 are best read together. Although the composite adverse outcome was numerically higher in the appendectomy group, the only robust adjusted predictors were culture-confirmed infection and lower gestational age, both of which are among the most consistently reported determinants of severe morbidity and mortality in very preterm infants [8,9,16,17,18,34]. The association between late-onset sepsis and bronchopulmonary dysplasia in our cohort is concordant with nationwide cohort evidence that infection contributes to BPD risk [10], and the link between prolonged empirical antibiotic exposure and necrotizing enterocolitis echoes earlier work showing harm from extended early antibiotics in extremely low-birth-weight infants [19]. The resource-utilization differences (Table 5)—longer length of stay, more ventilator and central-line days, and greater antimicrobial exposure—are the most consistent findings in this study and are consistent with the established downstream cost of late-onset infection and respiratory morbidity in this population [9,16,17,34]. These resource differences also provide the most parsimonious explanation for the exploratory pathway analyses in Table 9, in which length of stay and ventilation co-varied with caregiver symptom scores.

The caregiver findings (Table 6, Figure 4) are consistent with, and extend, a large body of work documenting elevated anxiety, depression, and reduced quality of life among NICU parents [20,21,25,26,27,28]. The magnitude of the PHQ-9 and GAD-7 differences we observed is comparable to that reported in meta-analytic and scoping summaries of NICU parental distress [20,28,35], and the worse scores among caregivers in the open- and negative-appendectomy subgroups are plausibly explained by the combination of a more difficult maternal surgical experience and a more complicated, longer neonatal course [24,29,30]. The subgroup gradients in Table 8 and Figure 2—less favorable signals for open surgery, negative histopathology, and third-trimester operation—parallel obstetric literature reporting worse outcomes for later-gestation and negative appendectomy [4,22,23,29,30,31], although the small subgroup sizes and selection by gestational timing preclude any causal reading. Contrasting evidence should also be acknowledged: some series report broadly favorable neonatal outcomes after appendectomy in pregnancy when surgery is timely and uncomplicated [5,22,36], and meta-analytic comparisons of laparoscopic and open approaches have not uniformly demonstrated neonatal benefit of one technique over the other [5,37]. Our exploratory subgroup signals, therefore, add to but do not resolve this mixed literature, and they should be tested in larger multicenter cohorts with prespecified subgroups and competing-risk analyses [38].

The caregiver findings also require cautious clinical interpretation. PHQ-9, GAD-7, and HADS are screening and symptom-severity instruments, not diagnostic interviews. Consequently, the results should be described as depressive symptoms, anxiety symptoms, and screening-based psychological distress rather than clinical depression or anxiety disorders. Maternal surgery during pregnancy, postoperative recovery, obstetric complications, prior mental health, social support, and socioeconomic factors could all contribute to discharge-time scores independently of the infant’s NICU course.

From a microbiological perspective, this study provides only a broad description of culture-confirmed bacterial and *Candida* infections. We did not collect isolate-level antimicrobial resistance profiles, molecular virulence markers, colonization surveillance cultures, or source-attribution data; therefore, the results cannot determine whether maternal appendectomy exposure was associated with specific pathogen lineages, multidrug-resistant organisms, or distinct transmission pathways. Future multicenter studies should integrate microbiology, antimicrobial exposure, central-line practices, and infection-prevention data to clarify whether the numerical infection signal observed here reflects patient vulnerability, device exposure, institutional ecology, or residual confounding.

### 4.2. Study Limitations

This study has several limitations that should be considered when interpreting the findings. First, as a single-center tertiary referral study, generalizability is constrained by local referral patterns, surgical practice, neonatal infection ecology, and NICU care protocols. Second, the cohort size limited precision for uncommon outcomes and for subgroup, mediation, and survival estimates. Third, despite frequency-matching and multivariable adjustment, residual confounding from unmeasured maternal disease severity, socioeconomic status, perioperative management, obstetric indication, and care-process variables cannot be excluded. Fourth, caregiver psychological outcomes were measured at a single pre-discharge timepoint, whereas prior work indicates that parental distress may evolve over time after NICU discharge [25,26,27,28]. Finally, the mediation and subgroup analyses should be interpreted as hypothesis-generating rather than causal. Additional limitations include the lack of a complete delivery and appendectomy denominator, possible selection effects from the minimum observation requirement, limited information on spontaneous versus medically indicated preterm birth pathways, absence of pathogen resistance or virulence typing, and the possibility of type I error inflation because multiple secondary and exploratory comparisons were performed. The relatively large number of appendectomy-exposed preterm infants accrued over a 34-month window should be interpreted in light of the center’s role as a regional tertiary referral hub. Because mothers experiencing appendicitis during pregnancy—particularly complicated or later-gestation cases—were preferentially transferred to and delivered at this institution, the exposed cohort is enriched relative to the source population, and the exposed-to-control ratio observed here does not reflect the true population incidence of preterm birth after appendectomy. This referral enrichment may bias the exposed group toward greater clinical complexity and could inflate apparent between-group differences in infection, resource utilization, and caregiver distress. The institutional background figures reported in the Methods are administrative approximations and were not prospectively audited, so the magnitude of selection bias cannot be precisely quantified; the comparative estimates should therefore be regarded as internally valid within this exposure-enriched cohort but not directly generalizable to unselected populations.

## 5. Conclusions

This prospective cohort study provides evidence that preterm infants born after maternal appendectomy during pregnancy experience a measurably different neonatal trajectory, suggesting that preterm infants born after maternal appendectomy during pregnancy may have a more resource-intensive neonatal trajectory compared with gestational-age-comparable preterm infants born without antecedent non-obstetric surgery. The appendectomy group demonstrated higher rates of culture-confirmed neonatal infection, longer NICU stays, greater ventilatory and antimicrobial burden, and more pronounced composite morbidity showed non-significant numerical increases in culture-confirmed neonatal infection and composite morbidity, together with significantly longer NICU stays and greater ventilatory and antimicrobial burden, with the adverse signal concentrated among with exploratory adverse signals concentrated among neonates whose mothers underwent open appendectomy, had negative histopathology, or had surgery in the third trimester. Equally importantly, caregivers of appendectomy-group infants reported significantly worse mental health at discharge, with depression and anxiety scores approaching or exceeding clinical thresholds for intervention—a burden substantially mediated through prolonged hospitalization. Caregivers of appendectomy-group infants reported higher discharge-time depressive and anxiety symptom scores; these screening-based findings may reflect both the infant’s hospitalization and the maternal surgical/obstetric experience. These findings have three practical implications. First, neonatal teams receiving preterm infants born after maternal appendectomy may consider careful infection surveillance while recognizing that the infection difference was not statistically significant. Second, the exploratory subgroup pattern should not be interpreted as proof that laparoscopy itself improves neonatal outcomes, although it remains consistent with guideline-supported minimally invasive approaches when clinically feasible, while the less favorable exploratory findings after negative appendectomy underscore the value of rigorous diagnostic imaging to minimize non-therapeutic surgery. Third, the higher discharge-time caregiver symptom scores may support consideration of caregiver psychological symptom screening within NICU discharge planning and could inform the design of future studies, particularly for families exposed to maternal surgical emergencies and prolonged neonatal hospitalization. Given the observational design and the non-significant primary infection outcome, these observations are hypothesis-generating and are not intended as clinical practice recommendations.

## Figures and Tables

**Table 1:** Baseline neonatal and perinatal characteristics by study group.

Variable	Appendectomy (*n* = 54)	Control (*n* = 67)	*p*-Value
GA, weeks	31.4 ± 2.3	30.8 ± 2.1	0.142
Birth weight, g	1612.3 ± 421.7	1547.8 ± 398.4	0.389
Male sex	25 (46.3%)	33 (49.3%)	0.743
Cesarean delivery	34 (63.0%)	38 (56.7%)	0.479
Antenatal steroids (complete)	37 (68.5%)	49 (73.1%)	0.576
Multiple gestation	4 (7.4%)	6 (9.0%)	0.758
PROM	7 (13.0%)	16 (23.9%)	0.127
Clinical chorioamnionitis	11 (20.4%)	8 (11.9%)	0.203
SGA	6 (11.1%)	9 (13.4%)	0.695
SNAPPE-II score	19.7 ± 6.8	18.4 ± 7.1	0.317
5 min Apgar < 7	8 (14.8%)	12 (17.9%)	0.646
Surfactant administration	22 (40.7%)	31 (46.3%)	0.545

Data are mean ± SD or *n* (%). Abbreviations: GA, gestational age; PROM, premature rupture of membranes; SGA, small for gestational age; SNAPPE-II, Score for Neonatal Acute Physiology with Perinatal Extension II. *p*-values from an independent *t*-test (continuous) or χ^2^/Fisher’s exact test (categorical).

**Table 2:** Appendectomy surgical and histopathological characteristics (appendectomy group, *n* = 54).

Variable	LA (*n* = 31)	OA (*n* = 23)	*p*-Value
GA at surgery, weeks	19.8 ± 7.6	24.3 ± 8.9	0.048 *
Surgery in 1st trimester	6 (19.4%)	2 (8.7%)	0.448
Surgery in 2nd trimester	18 (58.1%)	11 (47.8%)	0.449
Surgery in 3rd trimester	7 (22.6%)	10 (43.5%)	0.098
Surgery-to-delivery, days	97.3 ± 39.8	72.1 ± 43.6	0.028 *
Inflamed appendix	23 (74.2%)	15 (65.2%)	0.468
Negative appendectomy	8 (25.8%)	8 (34.8%)	0.468
Perforated appendicitis	3 (9.7%)	4 (17.4%)	0.440
Wound infection	0 (0.0%)	2 (8.7%)	0.176
Operative time, min	48.6 ± 14.3	57.9 ± 18.7	0.043 *
Hospital stay (surgery), days	2.8 ± 1.1	4.3 ± 1.9	0.001 *

Data are mean ± SD or *n* (%). Abbreviations: GA, gestational age; LA, laparoscopic appendectomy; OA, open appendectomy. *p*-values from an independent *t*-test or Fisher’s exact test comparing LA vs. OA subgroups; *—statistically significant.

**Table 3:** Neonatal infection rates and microbiology by study group. (Primary outcome).

Variable	Appendectomy (*n* = 54)	Control (*n* = 67)	*p*-Value
Any culture-confirmed infection	19 (35.2%)	14 (20.9%)	0.078
Bacterial sepsis	14 (25.9%)	11 (16.4%)	0.195
Candidemia	5 (9.3%)	3 (4.5%)	0.465
Early-onset sepsis (<72 h)	2 (3.7%)	1 (1.5%)	0.587
Late-onset sepsis (≥72 h)	17 (31.5%)	13 (19.4%)	0.125
DOL at first positive culture	11.4 ± 4.7	13.8 ± 5.1	0.152
Time to effective therapy, h	14.7 ± 6.8	12.9 ± 5.4	0.413
CoNS	5 (9.3%)	4 (6.0%)	0.514
Gram-negative organisms	7 (13.0%)	5 (7.5%)	0.325
*Candida* spp.	5 (9.3%)	3 (4.5%)	0.465
Polymicrobial	2 (3.7%)	2 (3.0%)	1.000
Recurrent infection (≥2 episodes)	6 (11.1%)	3 (4.5%)	0.176

Data are mean ± SD or *n* (%). Abbreviations: CoNS, coagulase-negative staphylococci; DOL, day of life. *p*-values from an independent *t*-test (continuous) or χ^2^/Fisher’s exact test (categorical).

**Table 4:** Major neonatal clinical outcomes by study group. (Secondary outcome).

Outcome	Appendectomy (*n* = 54)	Control (*n* = 67)	*p*-Value
Composite adverse outcome *	21 (38.9%)	17 (25.4%)	0.107
In-hospital mortality	4 (7.4%)	6 (9.0%)	0.758
Moderate–severe BPD	16 (29.6%)	14 (20.9%)	0.265
NEC ≥ stage II	6 (11.1%)	3 (4.5%)	0.176
Severe IVH (grade III–IV)	7 (13.0%)	5 (7.5%)	0.325
ROP requiring treatment	8 (14.8%)	5 (7.5%)	0.197
Periventricular leukomalacia	3 (5.6%)	2 (3.0%)	0.653
Culture-confirmed meningitis	3 (5.6%)	1 (1.5%)	0.322

Data are *n* (%). * Composite adverse outcome defined as any of: in-hospital mortality, moderate–severe BPD, NEC Bell stage ≥ II, severe IVH grade III–IV, ROP requiring treatment, or PVL. *p*-values from χ^2^ or Fisher’s exact test.

**Table 5:** NICU resource utilization by study group. (Secondary outcome).

Variable	Appendectomy (*n* = 54)	Control (*n* = 67)	*p*-Value
Length of stay, days	47.3 ± 14.8	41.2 ± 12.6	0.014 *
Invasive ventilation, days	21.6 ± 9.7	17.8 ± 8.4	0.022 *
Total respiratory support, days	36.8 ± 15.1	31.4 ± 13.2	0.036 *
Central-line days	21.4 ± 10.3	17.6 ± 8.7	0.028 *
Parenteral nutrition, days	17.8 ± 8.6	15.1 ± 7.3	0.063
Systemic antibiotic days	16.4 ± 7.9	13.2 ± 6.1	0.011 *
Systemic antifungal days	3.1 ± 5.4	1.4 ± 3.2	0.029 *
Blood transfusions	2.3 ± 1.8	1.7 ± 1.4	0.043 *

Data are mean ± SD. *p*-values from an independent *t*-test or Mann–Whitney U test. Significant *p*-values (*p* < 0.05) are marked with *; skewed resource variables were compared using non-parametric tests when Shapiro–Wilk testing indicated non-normality. Because several resource variables were right-skewed, medians (interquartile range) are also provided for the appendectomy versus control groups: length of stay 45 (37–56) vs. 39 (32–48) days; invasive ventilation 20 (14–28) vs. 16 (11–23) days; total respiratory support 35 (25–46) vs. 29 (21–40) days; central-line days 20 (13–28) vs. 16 (11–23); parenteral nutrition 16 (11–23) vs. 14 (9–20) days; systemic antibiotic days 15 (10–21) vs. 12 (8–17); systemic antifungal days 0 (0–4) vs. 0 (0–1); blood transfusions 2 (1–3) vs. 1 (1–2). *p*-values for these variables are from Mann–Whitney U tests.

**Table 6:** Caregiver-reported psychological outcomes at discharge by study group. (Secondary outcome).

Measure (at Discharge)	Appendectomy (*n* = 54)	Control (*n* = 67)	*p*-Value
SF-36 PCS	41.3 ± 6.8	44.1 ± 7.4	0.032 *
SF-36 MCS	39.2 ± 7.1	43.6 ± 7.8	0.002 *
WHOQOL-BREF Physical	53.4 ± 7.6	56.8 ± 8.3	0.018 *
WHOQOL-BREF Psychological	52.8 ± 8.4	57.1 ± 7.9	0.004 *
WHOQOL-BREF Social	59.4 ± 9.1	62.3 ± 8.6	0.074
WHOQOL-BREF Environmental	61.2 ± 7.8	65.1 ± 6.9	0.004 *
HADS-Anxiety	11.6 ± 3.4	9.7 ± 3.1	0.002 *
HADS-Depression	10.8 ± 3.1	8.9 ± 2.8	0.001 *
PHQ-9	12.4 ± 4.3	9.6 ± 3.8	<0.001 *
GAD-7	11.1 ± 3.6	8.9 ± 3.3	0.001 *
Body Image Scale	8.7 ± 3.1	6.8 ± 3.4	0.002 *

Data are mean ± SD. Abbreviations: BIS, Body Image Scale; GAD-7, Generalized Anxiety Disorder-7; HADS, Hospital Anxiety and Depression Scale; MCS, Mental Component Summary; PCS, Physical Component Summary; PHQ-9, Patient Health Questionnaire-9; SF-36, 36-Item Short Form Survey; WHOQOL-BREF, World Health Organization Quality of Life—BREF. Significant *p*-values (*p* < 0.05) are marked with *; these scales were used as screening/symptom instruments, not diagnostic interviews. Respondent was the mother in 44/52 (84.6%) appendectomy families and 52/64 (81.3%) control families; the remainder were completed by another primary caregiver (predominantly the father). Valid responses per scale: appendectomy group 49–52 of 54 (missing 2–5); control group 61–65 of 67 (missing 2–6). Item-level missing data were handled by complete-case analysis for each scale.

**Table 7:** Multivariable logistic regression: predictors of composite adverse neonatal outcome. (Exploratory analysis).

Predictor	aOR	95% CI	*p*-Value	Wald χ^2^
Appendectomy group (ref: control)	2.14	0.96–4.78	0.063	3.47
GA per additional week	0.71	0.58–0.87	0.001 *	10.82
SNAPPE-II per 5-point increase	1.34	1.06–1.69	0.014 *	6.08
Culture-confirmed infection	3.27	1.41–7.58	0.006 *	7.62
Open appendectomy (ref: LA/control) ^a^	2.89	1.12–7.46	0.028 *	4.84
Negative appendectomy (ref: inflamed/control) ^a^	3.41	1.18–9.86	0.023 *	5.16
Surgery-to-delivery interval (per 30 days) ^a^	0.82	0.68–0.99	0.041 *	4.17
Male sex	1.28	0.59–2.78	0.534	0.39

Abbreviations: aOR, adjusted odds ratio; CI, confidence interval; GA, gestational age; LA, laparoscopic appendectomy; SNAPPE-II, Score for Neonatal Acute Physiology with Perinatal Extension II. Model AUC = 0.812; Hosmer–Lemeshow *p* = 0.384. Appendectomy-specific indicators were exploratory and coded as absent in controls; results should be interpreted cautiously because of the limited events per variable. The composite outcome included 38 events. Group assignment, gestational age, SNAPPE-II score, culture-confirmed infection, and sex constitute the simplified main model fitted in the full cohort (*n* = 121). Predictors marked with a superscript “a” (open appendectomy, negative appendectomy, and surgery-to-delivery interval) are undefined in controls and are reported from a separate exploratory model restricted to the appendectomy group (*n* = 54); they are not part of the whole-cohort model and are shown together here only for compactness. Given the low events-per-variable ratio, all adjusted estimates are hypothesis-generating; *—statistically significant.

**Table 8:** Subgroup analysis of neonatal outcomes and caregiver distress by appendectomy characteristics. (Exploratory analysis).

Subgroup	Infection Rate (%)	Composite Adverse (%)	LOS (Days)	PHQ-9 Score	*p* (Interaction)
LA (*n* = 31)	29.0%	32.3%	43.7 ± 13.1	11.2 ± 3.9	—
OA (*n* = 23)	43.5%	47.8%	52.1 ± 15.6	14.1 ± 4.3	0.037 *
Inflamed (*n* = 38)	31.6%	34.2%	45.2 ± 13.8	11.8 ± 4.1	—
Negative (*n* = 16)	43.8%	50.0%	52.4 ± 16.1	13.9 ± 4.4	0.024 *
1st trimester (*n* = 8)	12.5%	12.5%	37.4 ± 9.8	9.8 ± 3.1	—
2nd trimester (*n* = 29)	34.5%	37.9%	46.8 ± 13.7	12.3 ± 4.2	0.118
3rd trimester (*n* = 17)	47.1%	52.9%	53.6 ± 15.9	14.6 ± 4.1	0.008 *
Control (*n* = 67)	20.9%	25.4%	41.2 ± 12.6	9.6 ± 3.8	ref.

Data are *n* (%) or mean ± SD. *p* (interaction) tests whether the appendectomy characteristic modifies the outcome compared with the control group. Abbreviations: LA, laparoscopic appendectomy; LOS, length of stay; OA, open appendectomy; PHQ-9, Patient Health Questionnaire-9. The *p* (interaction) column refers to exploratory interaction testing for the corresponding appendectomy characteristic in models emphasizing length of stay; subgroup infection and composite outcomes are descriptive; *—statistically significant.

**Table 9:** Mediation analysis: indirect pathways between surgical exposure, neonatal burden, and caregiver outcomes. Estimates describe statistical association along hypothesized pathways and should not be interpreted as established causal mediation).

Mediation Path	Total Effect (c)	Direct Effect (c’)	Indirect Effect (ab)	% Mediated	Sobel *p*
Appendectomy → Infection → Composite	0.136	0.084	0.052	38.2%	0.031 *
Appendectomy → LOS → PHQ-9	2.81	1.47	1.34	47.7%	0.008 *
Appendectomy → Ventilation → SF-36 MCS	−4.42	−2.68	−1.74	39.4%	0.019 *
Infection → LOS → HADS-D	2.14	0.93	1.21	56.5%	0.003 *
Infection → Central-line days → Candidemia	0.048	0.021	0.027	56.3%	0.014 *
OA (vs. LA) → LOS → Composite	0.154	0.089	0.065	42.2%	0.042 *

Mediation analyses conducted using the Baron and Kenny framework with 5000 bootstrap resamples. Sobel *p* tests the significance of the indirect (mediated) pathway. Abbreviations: HADS-D, Hospital Anxiety and Depression Scale—Depression; LA, laparoscopic appendectomy; LOS, length of stay; MCS, Mental Component Summary; OA, open appendectomy; PHQ-9, Patient Health Questionnaire-9. Mediation analyses were exploratory pathway analyses; estimates should not be interpreted as causal effects; *—statistically significant.

## Data Availability

The data presented in this study are available on request from the corresponding author. The data are not publicly available due to privacy and ethical restrictions.
